# Test-Retest Reliability of Low-Cost Posturography for Assessing Postural Stability Control Performance during Standing

**DOI:** 10.1155/2021/9233453

**Published:** 2021-08-05

**Authors:** Sumet Heamawatanachai, Witawit Wiriyasakunphan, Kanokwan Srisupornkornkool, Chaiyong Jorrakate

**Affiliations:** ^1^Faculty of Engineering, Naresuan University, Phitsanulok 65000, Thailand; ^2^Faculty of Allied Health Sciences, Naresuan University, Phitsanulok 65000, Thailand

## Abstract

Postural stability control performance assessment is necessary in providing important information for individuals who are at risk of falling or who have balance impairment. Instrumented assessment is suggested as a valid and reliable test, but the cost and the difficulty of setup are significant limitations. The aim of this cross-sectional (test-retest reliability) study was to develop and determine the reliability of a low-cost posturography for assessing postural stability control performance during standing. The low-cost posturography was developed with four load cells and an acrylic platform. The center of pressure (COP) displacement and velocity were analyzed using written software. Test-retest reliability was performed with six different standing postural stability tests in twenty healthy volunteers on two different days. Intraclass correlation coefficient (ICC), standard error of measurement (SEM), coefficient of variation (CV), and Bland–Altman plot and limits of agreements (LOA) were used for analyses. The low-cost posturography was accurate (ICC = 0.99, *p* < 0.001; SEM = 0.003 cm) when compared to the true with calculated *X* and *Y* coordinates, with a moderate to excellent test-retest reliability for both COP displacement (ICCs ranged 0.62–0.91, *p* < 0.05; SEMs ranged 17.92–25.77%) and COP velocity (ICCs ranged 0.62–0.91, *p* < 0.05; SEMs ranged 18.09–27.69%) in all standing postural stability tests. Bland–Altman plots and LOAs suggested good agreement of tested parameters from the developed low-cost posturography between different days. In conclusion, the developed low-cost posturography had adequate reliability for assessing COP displacement and velocity during standing postural control stability performance tests.

## 1. Introduction

Postural control is an essential component of the motor control needed to achieve a body motion oriented to daily living environments [1, 2]. Postural control is governed by the central nervous system in order to purposefully accomplish target movements [2]. Postural control consists of two major components, including postural stability control (both static and dynamic movements) and postural equilibrium control [3, 4]. Postural stability control performance during static standing is a fundamental capability of humans, achieved by stabilizing the core of the body to efficiently move peripheral extremities.

Impairment of postural stability control not only causes ineffective motion but also leads people to become a burden; with a huge cost to healthcare services, wasted time, loss of opportunities and a poor quality of life [5, 6]. Problems with postural stability control are commonly found across a variety of age ranges, including children and adolescents [7, 8], adults [9] and the elderly [10, 11]. Exercise interventions have been utilized and suggested as an effective strategy to recover postural stability control performance [12–14]. Besides effective treatment, the assessment of postural stability control performance is also important, as it helps the clinician to monitor the progression of treatment and to establish an appropriate goal for postural stability control rehabilitation [15, 16]. Additionally, postural stability control assessments are suggested to be routinely applied to screen the elderly for early detection of a risk of falling [17].

Instrumented postural stability testing has been widely used for assessing postural stability control performance [15]. Instrumented testing with a force platform, posturography, and stabilography are frequently provided to assess postural stability control performance. With those instrumented tests, the center of pressure (COP) is a quantitative parameter which is usually used to quantify postural stability control performance [18, 19]. The subparameters of the COP usually reported in previous studies include position of the COP, root mean square (RMS) amplitude of the COP, total excursion of the COP, sway velocity of the COP, and sway area of the COP. The COP displacement (or the COP path length) and the COP velocity were suggested to be reliable and valid measures for determining postural stability control performance during standing [15, 18–21]. However, sophisticated postural stability assessment instruments are high in cost, complexity, and time-consumption related to settings. Furthermore, most of them were often furnished in research areas and could not be made easily accessible. Therefore, a number of studies have focused on developing accurate and reliable tools for determining postural stability control performance with lower cost equipment [22, 23]. In the last decade, several studies demonstrated that a Wii Balance Board^TM^ (WBB) with developed software was a valid and reliable tool for a low-cost postural stability control performance assessment [20, 22, 24–27]. The WBB seemed to be a suitable choice; however, the developed software from previous studies is not widely disseminated. In addition, the cost of the WBB is quite high and is generally considered to be inaccessible in clinical settings and to the low-income population. Therefore, the current study aimed to report the development of a low-cost, accurate, and reliable posturography, evaluating COP time-domain parameters. It was hypothesized that the developed posturography would display accurate measurement and reliable test-retest reliability. This low-cost posturography could offer easier access and more user friendly use in clinical settings.

## 2. Materials and Methods

The current study utilized a cross-sectional research design with test-retest reliability. A low-cost posturography was developed as a prototype named “Standing Balance Assessment Posturography (SBAP).” The SBAP was purposefully used to analyze the COP time-domain parameters (COP displacement and velocity) during standing. All testing procedures were approved by the Naresuan University Institutional Review Board (IRB no. P10186/63). All volunteers signed an informed consent before participating in the current study.

### 2.1. Participants

Participants were recruited in Naresuan University area through purposive sampling, using flyers, posters, and personal contacts. The number of participants (20 volunteers) was determined according to a guideline for sample size estimation for analysis with the intraclass correlation coefficient (ICC) (two observations per subject, 90% power and ICC = 0.6) [28]. Healthy young adult volunteers (10 male and 10 female) aged 10–25 years old with no history of back and lower extremity arching and no musculoskeletal and neurological problems were recruited. Volunteers with apparent standing balance disturbance, other problems related to postural control disability, a history of recurrent ankle sprain, a history of serious traumatic injury to the back and lower extremities, and undertaking any exercise or sports training programs were excluded. All volunteers were asked not to sleep less than 6 hours per night and not to use medications or consume alcohol which would affect postural stability control performance prior to participating in this study. In female volunteers, testing procedures were not conducted during their menstruation period or during pregnancy.

### 2.2. Instruments and System Overview

**Figure 1:**
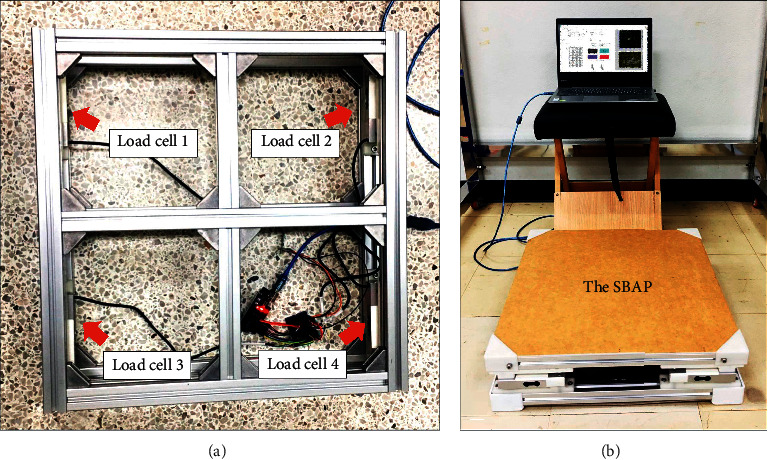
The standing balance assessment posturography (SBAP): (a) position of load cells; (b) overview of SBAP.

The SBAP (Figure 1) consisted of an acrylic platform (50 cm width x 50 cm length x 0.5 cm thickness) with four parallel beam load cells (each of 100 kg rated capacity, OEM) embedded under the four corners of platform. The load cells were connected with signal amplifiers (model Hx711) and a microcontroller (Arduino Uno). The acrylic platform was mounted on an aluminium frame (50 cm width x 50 cm length x 15 cm height) which was glued to a nonslip material on the bottom surface. The SBAP was interfaced with a laptop computer (Lenovo Intel® Core™ i5-8250U, CPU 1.6 GHz) via a USB cable connector. The weight of SBAP was approximately 7 kg.

**Figure 2:**
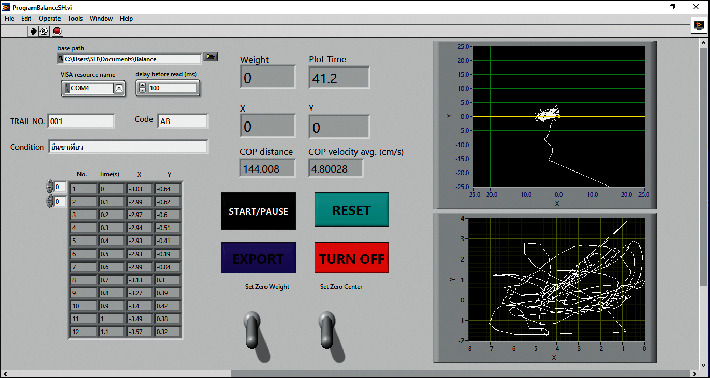
Display of the developed software with SBAP.

The custom-made software for calculating the COP displacement and velocity was developed and written in LabVIEW. The COP displacement was referred to as a whole distance of the COP over the test duration (unit of measurement: centimeter, cm). The COP velocity was derived from the COP displacement over time (unit of measurement: centimeter per second; cm/second). The signals from the four parallel beam load cells were delivered and converted to digital information by the written software. The sampling frequency was recruited at 10 Hz by the software with no data filtering. The signals from the load cells were processed and computed to get the total weight press on the platform along with the COP location (in the form of *X* and *Y* coordinates). The COP displacements were calculated from the total distance of change in the COP location over the testing duration. The COP velocities were calculated from the COP displacement divided by the testing duration. After clicking the start button of the developed software, SBAP started recording the input information for 40 seconds and then stopped automatically. The COP parameters were then processed and calculated during the middle 30 seconds. The COP displacement, the COP velocity, and a graphical real-time COP trajectory were presented on the laptop's screen (Figure 2).

**Figure 3:**
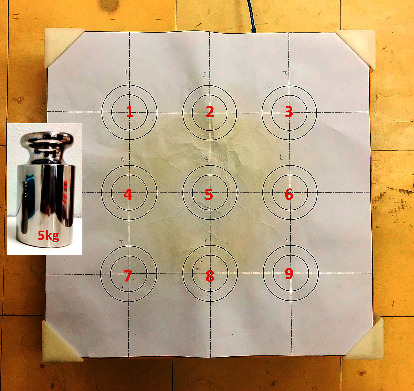
Nine positions and a 5 kg weight for testing SBAP accuracy.

Before conducting postural stability control performance tests with SBAP, the accuracy of SBAP was initially tested with a standard 5 kg weight placed sequentially in nine positions over the platform (Figure 3). The 5 kg weight was placed 5 times repeatedly in each position. The actual or true positions on the platform and the 45 calculated positions (9 positions x 5 times) from the software were then analyzed for the accuracy of the *X* and *Y* coordinates of the system.

### 2.3. Data Collection

After volunteers had signed an informed consent, they were screened according to the inclusion and exclusion criteria. Volunteers who passed the criteria were tested for their dominant leg by performing three activities: kicking a ball, writing their names on the floor, and picking up an object. The dominant leg was identified if they used the same leg to perform at least 2 activities.

All measurements were conducted at a single site, the Faculty of Allied Health Sciences, Naresuan University, Thailand. The laboratory room was silent and no other activities were allowed to avoid distraction. Initially, volunteers were asked to wear a comfortable shirt, short pants, and be bare foot. To prevent falling, volunteers wore a full body harness with nylon rope slings firmly suspended from the supporting frame. All volunteers were introduced to the testing protocols and were allowed to practice until they became familiar with the testing procedure.

**Figure 4:**
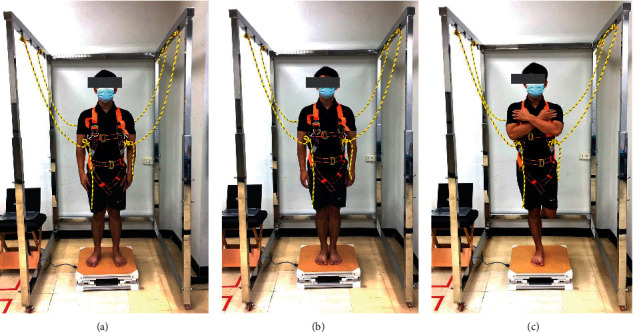
Standing postural stability control tests: (a) double leg stance with feet shoulder width apart, (b) double leg stance with feet together, and (c) single leg stance.

Postural stability control performance was tested via standing balance assessments. The standing balance assessments were varied visual inputs and bases of support to challenge postural control ability. Standing balance assessments were tested in 6 different conditions, and each condition was performed thrice. The COP trajectory real-time display was eliminated from volunteers during the tests. Successful trials were affirmed when the volunteer could stand without swaying or falling and did not open their eyes during the eyes closed condition. If an unsuccessful trial occurred, the volunteer was asked to perform repeatedly until 3 successful trials in each condition were completed. The 6 different standing postural stability control performance test conditions were as follows (Figure 4).

- Double leg stance with feet shoulder width apart and eyes open (DLS-SW-EO), arms by their sides, the distance between feet was recorded
- Double leg stance with feet shoulder width apart and eyes closed (DLS-SW-EC), arms by their sides, the distance between feet was recorded
- Double leg stance with feet together and eyes open (DLS-FT-EO), arms by their sides
- Double leg stance with feet together and eyes closed (DLS-FT-EC), arms by their sides
- Single leg stance with eyes open (SLS-EO), other leg bent at 90 degrees of knee flexion toward the back, arms crossed on their chest
- Single leg stance with eyes closed (SLS-EC), other leg bent at 90 degrees of knee flexion toward the back, arms crossed on their chest

The testing conditions were randomly assigned for each volunteer. The volunteers were asked to stand still in the middle of SBAP for over 40 seconds in each test. Two minutes rest or longer was allowed between trials or until the volunteers had no fatigue or tiredness before starting the new trial or condition. The SBAP was set at zero shift before collecting data in each trial. All testing procedures were performed twice with identical procedures for all volunteers in two different sessions. The second session was tested 24 hours after the first session. Each session took place over approximately 45 minutes. All measurements in the two sessions were conducted by the same tester who was appropriately instructed and trained in all testing protocols. The tester separately recorded and exported the testing parameters of individual volunteers after completing the testing procedures in each session. All testing parameters were then analyzed with statistical software by another researcher.

### 2.4. Statistical Analysis

#### 2.4.1. Accuracy of SBAP

The mean differences between the true and calculated *X* and *Y* coordinates from the nine positions over the platform (*N* = 45) were determined for accuracy using an intraclass correlation coefficient (two-way random, absolute agreement, single measure) [29] and a standard error of measurement (SEM). The SEM was analyzed using the equation “SEM = SD ^*∗*^ (square root of 1-ICC)” [25, 30].

#### 2.4.2. Test-Retest Reliability of SBAP during Postural Stability Control Performance Tests in Six Conditions

The characteristics of volunteers were descriptively reported. The mean of the COP displacements and the COP velocities during the six testing conditions in session 1 and 2 were descriptively demonstrated. Scatter plots were primarily checked for linearity of COP displacements and velocities between session 1 and 2. Afterward, test-retest reliability of the postural stability tests with SBAP were analyzed from different days (sessions 1 and 2) using the intraclass correlation coefficient (ICC) (two-way mixed effect, consistency, average measure). The values of the ICC were qualitatively classified as displaying excellent (ICC >0.90), good (ICC between 0.75 and 0.90), moderate (0.50–0.75), and poor (ICC <0.50) reliability [31]. Additionally, a coefficient of variation (CV) [30] and SEM of the COP displacement and velocity in sessions 1 and 2 were also analyzed. Furthermore, Bland–Altman plots for the COP displacements and velocities were also graphically displayed showing the agreement and systematic bias of each measurement between sessions with 95% limits of agreement (LOA). Statistical analysis was conducted with the Statistical Package for Social Sciences (SPSS). The *p* value was set at or less than 0.05 for all statistical analyses.

## 3. Results

### 3.1. Volunteers

Twenty young adult volunteers (age = 21.45 ± 0.59 years, weight = 53.21 ± 7.32, height = 165.50 ± 5.16 cm, and body mass index = 19.40 ± 2.34 kg/m^2^) were recruited in the current study. All volunteers were completely measured against the testing protocols in sessions 1 and 2. Physical and emotional changes which apparently disturbed postural stability control performance were not observed in all volunteers. No falling or serious adverse effects were detected throughout the study.

### 3.2. Accuracy of SBAP

Means and standard deviations of the differences between the true and calculated *X* and *Y* coordinates were −0.13 ± 0.22 cm and −0.12 ± 0.17 cm, respectively. The reliability coefficients from ICC analysis were 0.99 (95% confidence interval = 0.99–1.00, *p* < 0.001) for both *X* and *Y* coordinates. The SEMs of differences between true and calculated positions of *X* and *Y* coordinates were 0.022 cm and 0.017 cm, respectively. These results demonstrated high accuracy of SBAP to estimate the *X* and *Y* coordinates on platform.

### 3.3. Test-Retest Reliability of the Postural Stability Control Performance Test with SBAP in Six Conditions

**Figure 5:**
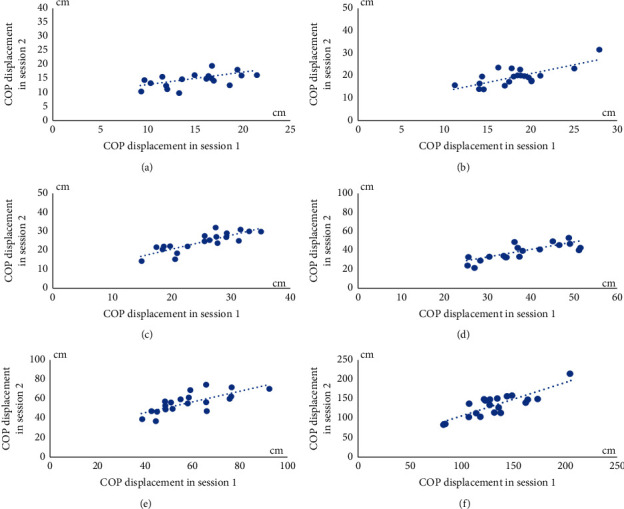
Scatter plots of COP displacements between sessions 1 and 2 from six testing conditions. (a) COP displacements during DLS-SW-EO. (b) COP displacements during DLS-SW-EC. (c) COP displacements during DLS-FT-EO. (d) COP displacements during DLS-FT-EC. (e) COP displacements during SLS-EO. (f) COP displacements during SLS-EC.

**Figure 6:**
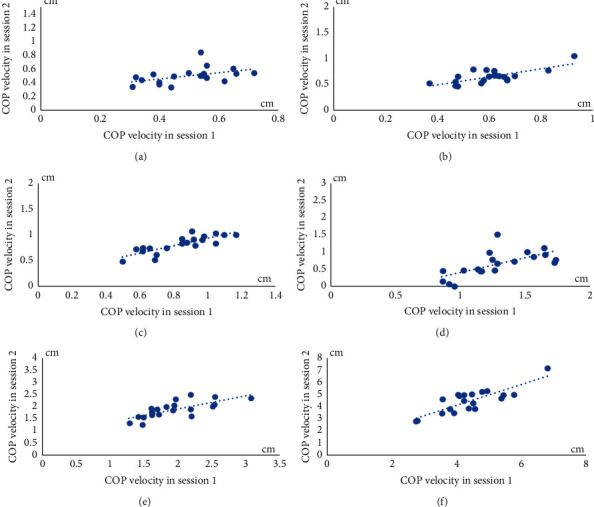
Scatter plots of COP velocities between sessions 1 and 2 from six testing conditions. (a) COP velocity during DLS-SW-EO. (b) COP velocity during DLS-SW-EC. (c) COP velocity during DLS-FT-EO. (d) COP velocity during DLS-FT-EC. (e) COP velocity during SLS-EO. (f) COP velocity during SLS-EC.

Scatter plots of both the COP displacement and velocities in six testing conditions showed the linearity relationships of parameters between sessions 1 and 2 (Figures 5 and 6). Means and standard deviations (SD) of the COP displacements and velocities in both sessions are demonstrated in Table 1. The results of the test-retest reliability of the postural stability control performance test with SBAP between sessions 1 and 2, expressed with ICC values, are given in Table 2. The results showed moderate to high test-retest reliability between sessions 1 and 2 in the six different conditions. A moderate test-retest reliability was found with the COP displacement and velocity in the double leg stance with feet shoulder width apart and eyes open condition. A good test-retest reliability was identified with the COP displacements and velocities in the double leg stance with feet shoulder width apart and eyes closed, double leg stance with feet together and eyes closed, and single leg stance with eyes open conditions. An excellent reliability was expressed with the COP displacement and velocity in the double leg stance with feet together and eyes open and single leg stance with eyes closed conditions.

The CV and SEM of the COP displacements and velocities in both sessions during the 6 testing conditions are reported in Table 3. In both sessions, the CV of the COP displacements ranged 17.92–25.77%, whereas the CV of the COP velocities ranged 18.09–27.69%. In each testing condition, consistent CV values were observed between sessions 1 and 2 for both the COP displacement and velocity.

**Figure 7:**
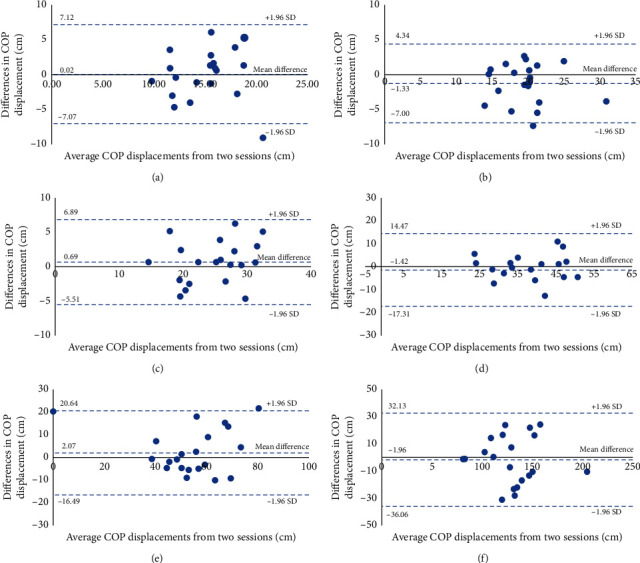
Bland–Altman plots and limits of agreements (LOAs) of COP displacements in sessions 1 and 2 in six testing conditions. (a) COP displacements during DLS-SW-EO. (b) COP displacements during DLS-SW-EC. (c) COP displacements during DLS-FT-EO. (d) COP displacements during DLS-FT-EC. (e) COP displacements during SLS-EO. (f) COP displacements during SLS-EC.

**Figure 8:**
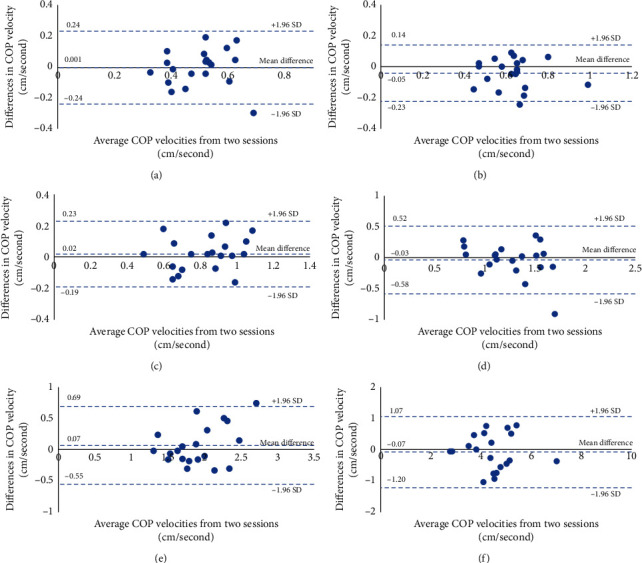
Bland–Altman plots and limits of agreements (LOAs) of COP velocities in sessions 1 and 2 in six testing conditions. (a) COP velocity during DLS-SW-EO. (b) COP velocity during DLS-SW-EC. (c) COP velocity during DLS-FT-EO. (d) COP velocity during DLS-FT-EC. (e) COP velocity during SLS-EO. (f) COP velocity during SLS-EC.

The SEM values were increased according to the level of difficulty of the testing conditions (from the easiest, condition 1, to the hardest, condition 6). Again, the SEM values were consistently observed between sessions 1 and 2 for both the COP displacement and velocity. The agreement of the COP displacements and velocities between session 1 and 2 in six testing conditions was demonstrated via Bland–Altman plots with LOAs (Figures 7 and 8). There were no obvious trends or systematic bias between the measurements in all testing conditions.

## 4. Discussion

In the study of reliability analysis, measurement errors can be attributed to three sources including rater, measuring instrument, and variability of the characteristics being measured [31]. Measurement errors were minimized by suitably designing the testing procedures and protocols. All measurements of testing protocols in the two sessions were conducted by the same well-trained tester with a clear procedure of testing protocols in order to control the measurement error from the rater. The tested parameters of individual volunteers were separately processed and exported after completing each session. Afterward, the tested parameters were analyzed by another researcher to reduce the rater's bias on data analyses. For eliminating the measurement error from measuring instrument, the low-cost posturography (SBAP) was initially tested for its accuracy by analyzing the differences between true and calculated *X* and *Y* coordinates before testing test-retest reliability in the young adult volunteers. The results of this study demonstrated that SBAP had excellent precision of calculated *X*, *Y* coordinates (ICC = 0.99, SEM <0.03 cm) when compared with the true positions on the platform. The protocols for postural control performance testing were appropriately designed as recommend by previous studies, including foot and leg positions, testing duration, repetitions of testing, visual acuity conditions, and random-order of the testing conditions [15, 18, 32, 33], in order to control the variability between sessions. Hence, the results of reliability analyses will mostly reflect the consistency of the postural stability control performance testing with SBAP.

In the current study, only one group of volunteers was measured twice for COP displacements and velocities. Young healthy adult volunteers who had no significant physical factors affecting postural stability control performance were recruited (both males and females in equal number). All volunteers were asked to sleep sufficiently and not to use medications or alcohol which would affect postural stability control prior to joining the testing protocols throughout the study. The interval durations between sessions 1 and 2 of all volunteers were approximately 24-hour apart. Female volunteers were not tested if they were in the menstruation period or pregnant. As mentioned above, the variability of the measurement scores caused by being tested participants would be probably controlled.

From testing conditions 1−6, the values of the COP displacement were gradually increased. These increasing values were also observed with the values of the COP velocity. The testing conditions were purposefully ranked according to the difficulty of the tests, from the wider base of support (BOS) with the presence of visual input to the steeper BOS and the absence of visual input. It was suggested that as the COP displacement and velocity increased, more postural stability was needed during quiet standing [21]. However, this concept might not be implied for all situations. Palmieri and colleagues [18] elucidated that COP displacement and velocity alone might not adequately explain the nature of postural stability control. Therefore, other parameters of the COP domain should be considered for postural stability control. Additionally, various factors affected the characteristics of postural stability control, such as testing conditions, testing protocol, assessment tools, and characteristics of subjects. As mentioned, the COP displacement and velocity may be better appropriated for individual longitudinal monitoring.

The gradual increases of the standard deviations of COP displacements and velocities were similarly observed in both sessions. This implies that the variability of the COP displacements and velocities increases as the difficulty of testing protocols increases (from testing conditions 1–6). However, most values of CVs of COP displacements and velocities in all testing conditions and sessions seem to be consistent across testing conditions and sessions (most of CV values were 20–30%). Therefore, it could be stated that the increased variability observed depended on the inherent difficulty of the testing conditions. The test-retest reliabilities of static postural control tests for both the COP displacement and velocity of SBAP were moderate to excellent. The SEM values of all measurements in this study were acceptable (less than 30%) [19, 32]. The agreement between measurements in all testing conditions for both the COP displacement and velocity was good, as demonstrated in the Bland–Altman plots and LOAs. Most differences between the 2 sessions of COP measurement with SBAP were within ±2 SD of the mean differences in both positive and negative directions. This indicated no significant bias between the measurements with SBAP from different sessions. Additionally, the values of the average COP displacement and velocity were consistent between sessions and was in a similar range to those from studies which evaluated the postural control performance during standing with the WBB and a laboratory-grade force platform [16, 19, 20, 25, 34], eventhough there were some discrepancies in testing parameters when compared to this study. Therefore, it could be stated that the developed low-cost posturography in this study had enough reliability for assessing postural stability control performance during standing.

The instrumented test for postural stability control performance is suggested to be better than the clinical tests, reporting more accurate and precise scores and providing more details related to biomechanical parameters. Nonetheless, the instrumented tests have rarely been used in clinical settings due to their difficulty of setup, heavy weight, and high cost. A number of studies have investigated the reliability and validity of a low-cost posturography [16, 20, 22, 24–27]. They proposed the Wii Balance Board^TM^ (WBB) as a low-cost posturography, which had moderate to excellent reliability and good validity in comparison with a laboratory-grade force platform. However, some technical limitations of the WBB were reported, including an inconsistent sampling rate and a poor signal to noise ratio, which may have impacted the analysis of the COP parameters [22]. Consequently, several studies tried to address those limitations by improving the input signals with low-pass filtering to attenuate noise [16, 20, 22, 24–27]; however, the technical details related to data acquisition and calculation of the written software were not precisely explained. Nowadays, the WBB has decreased in popularity, and eventhough the cost of the WBB has also decreased somewhat, its cost remains high for extensive use in clinical settings or even in home use. However, the pressure sensors used in SBAP are currently decreasing in price and are convenient in that they can be connected to various computer programs to effectively create the software for biomechanical analysis. Therefore, we decided to develop a low-cost posturography using simple load cells and platform. In future developments of the system, it would be possible to lower the cost of production and the weight of SBAP.

The limitations of this study were related to materials and external validity. The load cells were limited to 100 kg loaded. The sampling rate was low (10 Hz) with no data filtering, whereas previous studies used at least 40 Hz (for the WBB) or up to 200 Hz (for a laboratory-grade force platform) with low-pass filtering (less than 10 Hz) [15, 16, 20, 22, 24–27, 32]. However, the results of the recorded parameters still had sufficient reliability and were consistent with previous studies [16, 19, 20, 25, 34]. Volunteers in this study were symptom-free individuals, so the results could not be referred to another population.

In future studies, the accuracy of posturography should be tested with various weights and numbers of *X* and *Y* coordinates on the platform. Better load cells, data and signal processing, and more COP parameters should be developed and compared with a laboratory-grade force platform. Volunteers with balance disorders or an aging population should be recruited into these studies.

## 5. Conclusions

This study developed low-cost posturography (SBAP) for assessing postural stability control performance during standing. The results demonstrated that low-cost SBAP was accurate and had moderate to excellent test-retest reliability.

## Figures and Tables

**Table 1:** Means and standard deviations of COP displacements and velocities of six conditions in sessions 1 and 2.

Conditions	COP displacements		COP velocities	
	Session 1 (cm)	Session 2 (cm)	Session 1 (cm/second)	Session 2 (cm/second)
Double leg stance with feet shoulder width apart and eyes open	15.06 ± 3.42	15.04 ± 3.33	0.50 ± 0.11	0.50 ± 0.11
Double leg stance with feet shoulder width apart and eyes closed	18.21 ± 3.73	19.54 ± 3.98	0.61 ± 0.12	0.65 ± 0.13
Double leg stance with feet together and eyes open	25.18 ± 5.56	24.49 ± 4.86	0.84 ± 0.19	0.82 ± 0.16
Double leg stance with feet together and eyes closed	38.01 ± 8.29	39.43 ± 10.16	1.27 ± 0.28	1.30 ± 0.36
Single leg stance with eyes open	58.39 ± 13.55	56.31 ± 10.09	1.95 ± 0.45	1.88 ± 0.34
Single leg stance with eyes closed	132.21 ± 28.39	134.17 ± 29.23	4.41 ± 0.95	4.47 ± 0.97

**Table 2:** Results of test-retest reliability (intraclass correlation coefficients) for COP displacements and velocities between sessions 1 and 2 in six conditions.

Conditions	COP displacements		COP velocities	
	Reliability coefficients (95% confidence interval)	*P* value	Reliability coefficients (95% confidence interval)	*P* value
Double leg stance with feet shoulder width apart and eyes open	0.62 (0.05, 0.85)	0.02^*∗*^	0.62 (0.05, 0.85)	0.02^*∗*^
Double leg stance with feet shoulder width apart and eyes closed	0.85 (0.61, 0.94)	<0.001^*∗*^	0.85 (0.61, 0.94)	<0.001^*∗*^
Double leg stance with feet together and eyes open	0.91 (0.76, 0.96)	<0.001^*∗*^	0.91 (0.76, 0.96)	<0.001^*∗*^
Double leg stance with feet together and eyes closed	0.78 (0.44, 0.91)	0.001^*∗*^	0.78 (0.44, 0.91)	0.001^*∗*^
Single leg stance with eyes open	0.83 (0.56, 0.93)	<0.001^*∗*^	0.82 (0.56, 0.93)	<0.001^*∗*^
Single leg stance with eyes closed	0.91 (0.76, 0.96)	<0.001^*∗*^	0.91 (0.76, 0.96)	<0.001^*∗*^

^*∗*^Statistical significant at *p* < 0.05.

**Table 3:** Results of coefficients of variation (CV) and standard errors of measurement (SEM) of COP displacements (cm) and COP velocities (cm/second) of six conditions in sessions 1 and 2.

Conditions	COP displacements				COP velocities			
	Session 1		Session 2		Session 1		Session 2	
	CV (%)	SEM	CV (%)	SEM	CV (%)	SEM	CV (%)	SEM
Double leg stance with feet shoulder width apart and eyes open	22.71	2.11	22.14	2.05	22.00	0.07	22.00	0.07
Double leg stance with feet shoulder width apart and eyes closed	20.48	1.44	20.37	1.54	19.67	0.05	20.00	0.05
Double leg stance with feet together and eyes open	22.08	1.67	19.84	1.46	22.62	0.06	19.51	0.05
Double leg stance with feet together and eyes closed	21.81	3.89	25.77	4.77	22.05	0.13	27.69	0.17
Single leg stance with eyes open	23.21	5.59	17.92	4.16	23.08	0.19	18.09	0.14
Single leg stance with eyes closed	21.47	8.52	21.79	8.77	21.54	0.29	21.70	0.29

## Data Availability

The data used to support this study are available from the corresponding author upon request.
